# Preconditioning Strategies for Kidney Ischemia Reperfusion Injury: Implications of the “Time-Window” in Remote Ischemic Preconditioning

**DOI:** 10.1371/journal.pone.0124130

**Published:** 2015-04-16

**Authors:** Young Eun Yoon, Kwang Suk Lee, Kyung Hwa Choi, Kwang Hyun Kim, Seung Choul Yang, Woong Kyu Han

**Affiliations:** 1 Department of Urology, Urological Science Institute, Yonsei University College of Medicine, Seoul, Korea; 2 Department of Urology, CHA Bundang Medical Center, CHA University, Seongnam-si, Korea; 3 Department of Urology, Ewha Womans University Mokdong Hospital, Seoul, Korea; Centre for Inflammation Research, UNITED KINGDOM

## Abstract

Remote ischemic preconditioning (IP) is a potential renoprotective strategy. However, there has been no demonstrated result in large animals and the role of time window in remote IP remains to be defined. Using a single-kidney porcine model, we evaluated organ protective function of remote IP in renal ischemia reperfusion injury. Fifteen Yorkshire pigs, 20 weeks old and weighing 35–38 kg were used. One week after left nephrectomy, we performed remote IP (clamping right external iliac artery, 2 cycles of 10 minutes) and right renal artery clamping (warm ischemia; 90 minutes). The animals were randomly divided into three groups: control group, warm ischemia without IP; group 1 (remote IP with early window [IP-E]), IP followed by warm ischemia with a 10-minute time window; and group 2 (remote IP with late window [IP-L]), IP followed by warm ischemia after a 24-hour time window. There were no differences in serum creatinine changes between groups. The IP-L group had lower urinary neutrophil gelatinase-associated lipocalin than control and IP-E at 72 hours post-ischemia. At 72 hours post-ischemia, the urinary kidney injury molecule-1 (KIM-1) was lower in the IP-L group than in the control and IP-E groups, and the IP-L group KIM-1 was near pre-ischemic levels, whereas the control and IP-E group KIM-1 levels were rising. Microalbumin also tended to be lower in the IP-L group. Taken together, remote IP showed a significant reduction in renal injury biomarkers from ischemia reperfusion injury. To effectively provide kidney protection, remote IP might require a considerable, rather than short, time window of ischemia.

## Introduction

Ischemic preconditioning (IP) is a phenomenon that promotes tissue tolerance to ischemia reperfusion injury (IRI) by a brief period of ischemia and subsequent reperfusion before the ischemic injury. Since the concept of IP was first reported by Murry et al. [1] in 1986, several studies have assessed the organ protective effect of IP in various organs [2–5]. The mechanism of IP remains unclear. Nitric oxide [6], protein kinase C [7], MAP kinase and MAPKAP kinase 2 [8], mitochondria [9], and decreased capacity of immune cells [10] have all been suggested as potential mechanisms.

Many investigations have demonstrated that IP can reduce serum creatinine (SCr), blood urea nitrogen (BUN), and histological renal damage after renal IRI [11]. However, almost all previous reports have used rodent models, such as rat or mouse models [11]. It may be precarious to apply the results of these types of experimental animal models to humans because the renal physiology of humans is not similar that of rodents; it is more similar to the renal physiology of large animals, such as dogs or pigs [11,12]. Of the previous investigations in large animals, all were performed with local IP produced by clamping the renal artery itself [13–18]. However, it might be detrimental to apply local IP to humans by repeatedly clamping and declamping renal vessels. Moreover, the results from these studies were limited to reporting changes in only SCr or BUN.

Acute kidney injury (AKI) is usually estimated by measuring SCr changes. However, SCr does not reflect the severity of AKI exactly and promptly because SCr levels do not rise immediately after AKI [19]. By contrast, recently highlighted biomarkers, such as neutrophil gelatinase-associated lipocalin (NGAL), kidney injury molecule-1 (KIM-1), and microalbumin may be elevated in the serum or urine more quickly after an acute change of renal function [20]. Therefore, these biomarkers could more accurately estimate renal function in the early phase of AKI, and allow a more accurate prediction of the prognosis. Moreover, NGAL is a precocious biomarker of therapeutic response, because the decrease of NGAL precedes the decrease of SCr [21]. To our knowledge, few studies have heretofore reported the changes in renal injury biomarkers observed during experimental IP for renal IRI [22].

Although numerous studies have investigated IP and its effects in the last 2 decades, there has been no established or verified protocol of how to conduct the procedure. This could be one of the reasons that IP is not currently recommended for kidney transplantation or partial nephrectomy in humans. The lack of a standard protocol may be attributable to the wide variety of methods that have been used for various facets of IP, including the time window of ischemia. This time window is the time between the IP and the index ischemic event of the target organ; it has varied from 5 minutes [13] to 14 days [23] in the literature. No specific time window of ischemia has been recommended to maximize the organ protective of IP.

For the above reasons, we conducted the present study to demonstrate the organ protective function of remote IP on renal IRI using a large animal porcine model. We aimed to verify the protective effect of remote IP by measuring the changes in not only the SCr but also several renal injury biomarkers, such as NGAL, KIM-1, and microalbumin. In addition, we evaluated the effect on kidney IRI of the time window between the IP and index ischemia.

## Materials and Methods

### Ethics Statement

All animal procedures and experiments were performed according to a protocol approved by Institutional Animal Care and Use Committee, Yonsei University Health System (Permit number: 2012–0279), in accordance with the National Institutes of Health guidelines. Surgical procedures were performed under isoflurane anesthesia, and all efforts were made to minimize any distress.

### Animals and design of experiments

A total of 15 female Yorkshire pigs (*S*. *s*. *domesticus*), 20 weeks old and weighing 35–38 (median 37) kg, were used (XP bio, Anseong-si, Korea). They received standard laboratory food for 10 days before the experiments and were food deprived for 8 hours before the procedures. They were placed on the surgical table after premedication with xylazine (2 mg/kg) and tiletamine-zolazepam (5 mg/kg). The animals were intubated with an un-cuffed endotracheal tube and anesthesia was maintained with 2% isoflurane in 60% air and 40% oxygen. The pigs were mechanically ventilated with a tidal volume of 7 mL/kg, a positive end-expiratory pressure of 3 cm H_2_O, and a respiratory rate of 15/min. Body temperature was maintained at 37–39°C with an external heating system. A peripheral venous catheter in the cephalic vein was used for the continuous fluid infusion. Cardiac rhythm was monitored by an electrocardiogram and O2 saturation was monitored by a pulse oximetry.

**Fig 1:**
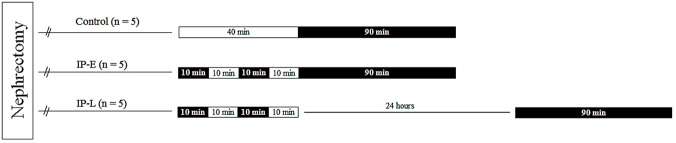
Protocol for the experiments used in ischemic preconditioning (40 minutes) and index ischemia (90 minutes). Black rectangles are the time zone of “clamping”, clear rectangles represent the “declamping”. See the Materials and Methods section for details. IP-E, remote ischemic preconditioning with early window; IP-L, remote ischemic preconditioning with late window.

To construct a single-kidney porcine model, we performed a laparoscopic left nephrectomy. After an adaption period of 1 week, IP and right renal artery clamping (warm ischemia) were performed as follows: IP was conducted by clamping the right external iliac artery for 2 cycles of 10 minutes ischemia followed by 10 minutes reperfusion, and warm ischemia was performed by clamping the remnant right renal artery for 90 minutes. The animals were randomly allocated into three experimental groups: control group (n = 5), 90 minutes of warm ischemia without IP; group 1 (n = 5, remote IP with early window [IP-E]) 20 minutes IP followed by 90 minutes warm ischemia after a 10-minute time window; and group 2 (n = 5, remote IP with late window [IP-L]), 20 minutes IP, followed by 24 hours as a time window of ischemia, which was subsequently followed by 90 minutes warm ischemia (Fig 1). These procedures were performed by two surgeons (W.K.H. and Y.E.Y.). All surgeries were performed laparoscopically, with the pneumoperitoneum maintained at 12 mmHg and three 5-mm ports used for instrumentation. Once the experiment was completed, the animals were euthanized with an intravenous injection of potassium chloride solution.

### Measurement of serum creatinine

SCr was measured at 6, 24, and 72 hours postoperatively after the ischemic event. After obtaining a 1 mL blood sample from the marginal auricular vein, we centrifuged the sample at 3,000 rpm for 5 minutes. Measurement of SCr was performed using a DRI-CHEM 4000 chemistry analyzer (Heska, Loveland, CO).

### Renal injury markers in urine

A urine sample was obtained by a veterinarian (he is not an author) who was unaware of the surgical results, using a nelaton catheter at 6, 24, and 72 hours, postoperatively. Obtained urine samples were encoded and stored in a -80°C deep freezer until ELISA analysis. All ELISA analyses were performed by a researcher (she is not an author) who was unaware of details of the experiment. NGAL was measured using a pig NGAL ELISA kit (Bioporto, Hellerup, Denmark). Microalbumin was measured using a pig microalbumin ELISA kit (KAMIYA, Seattle, WA). KIM-1 was measured using a porcine KIM-1 ELISA kit (MyBioSource Inc., San Diego, CA). All ELISA analyses were measured with a SpectraMax 190 (Molecular Devices, Sunnyvale, CA). Because hydration status can affect the concentration of these urinary markers, urinary creatinine was used as a normalization variable. Urine creatinine was measured by a HITACHI 7600 chemistry autoanalyzer (Hitachi, Tokyo, Japan).

### Statistical analysis

Data are shown as the median and interquartile range (IQR). For the analysis of SCr and kidney injury biomarkers, all variables were compared using the Kruskal-Wallis test and Mann-Whitney U-test. A p value ≤0.05 was considered statistically significant. Statistical analyses were performed using SPSS software (Statistical Product and Services Solutions, version 20.0, SPSS Inc., Chicago, Ill).

## Results

### Serum creatinine

**Fig 2:**
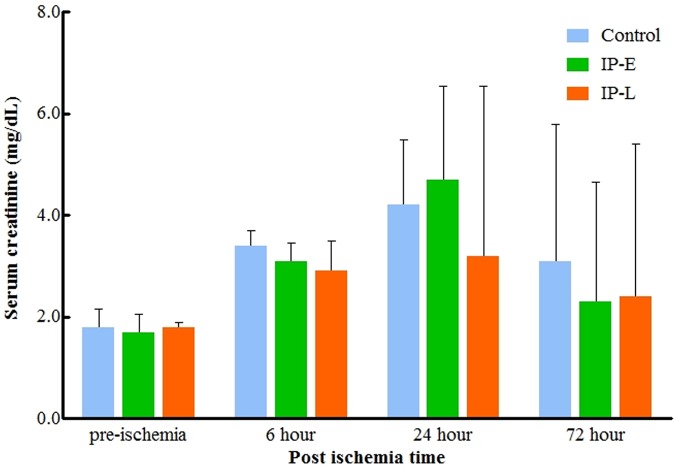
Changes in serum creatinine. There were no differences between groups throughout the whole experiment. SCr, serum creatinine; IP-E, remote Ischemic preconditioning with early window; IP-L, remote ischemic preconditioning with late window.

Before the left nephrectomy, all porcine SCr values were within the normal range (1.0–1.5 ng/dL), with a median of 1.2 (IQR 1.0–1.2) ng/dL. At 1 week after nephrectomy, the median pre-ischemic SCr of all groups was 1.8 (IQR 1.5–2.0) ng/dL (Fig 2). In all groups, the SCr was most up-regulated at 24 hours after the ischemic injury; however, the SCr at this time did not differ between the control, IP-E, and IP-L groups (4.2 [IQR 3.25–5.5] ng/dL, 4.7 [IQR 3.6–6.55] ng/dL, and 3.2 [IQR 2.9–6.55] ng/dL, respectively; p = 0.566). At 72 hours after ischemic injury, the SCr was still elevated above the pre-ischemic level in all groups, but again there was still no difference between the control, IP-E, or IP-L groups (3.1 [IQR 2.5–5.8] ng/dL, 2.3 [2.15–4.65] ng/dL, and 2.4 [1.95–5.4] ng/dL, respectively; p = 0.610).

### Urinary renal injury biomarkers

**Fig 3:**
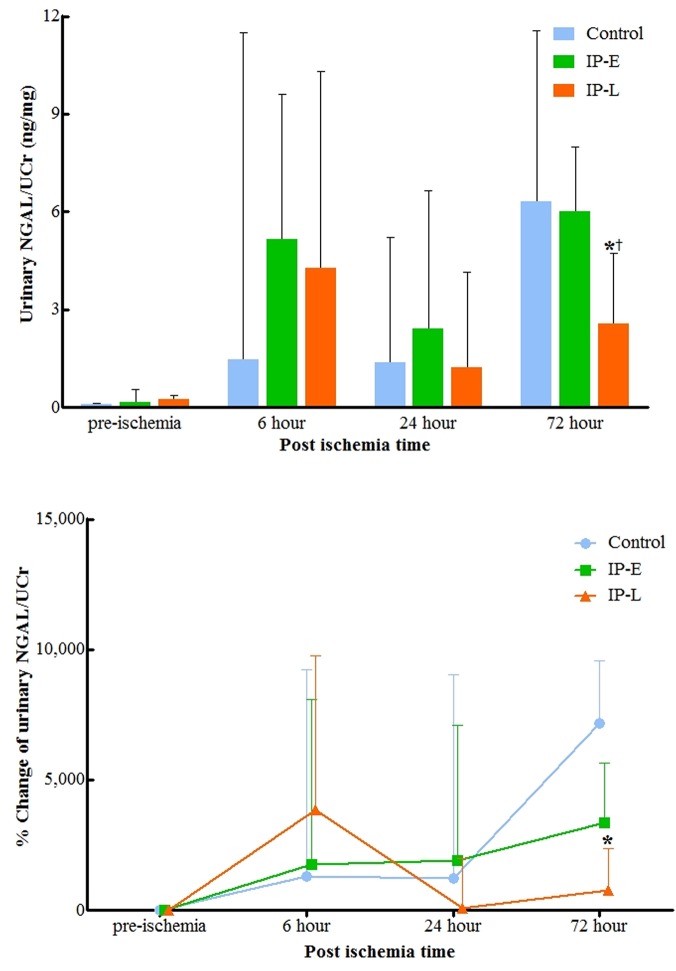
Changes in urinary NGAL (A) and urinary NGAL percent change from baseline (B) normalized to urinary creatinine. At 72 hours postoperatively, urinary NGAL was lower in the IP-L group than in the control and IP-E groups (p = 0.076 and 0.047, respectively). NGAL, neutrophil gelatinase-associated lipocalin; Ucr, urine creatinine; IP-E, remote ischemic preconditioning with early window; IP-L, remote ischemic preconditioning with late window. * p<0.05 vs. Control, ^†^ p<0.05 vs. IP-E.

Before index ischemia, the median urinary NGAL was 0.12 (IQR 0.1–0.26) ng/mg (Fig 3). At 6 hours after the ischemic injury, there was no difference in urinary NGAL levels between groups. At 24 hours after ischemia, the urinary NGAL was lower in the IP-L than in the control and IP-E groups; however, the difference did not reach the statistical significance (p = 0.602 and 0.347, respectively; Fig 3). At 72 hours post-ischemia, the NGAL was still lower in the IP-L group than in the other two groups (p = 0.076 and 0.047, respectively). There was no difference between the control and IP-E group NGAL levels throughout the observational period (up to 72 hours after IRI).

**Fig 4:**
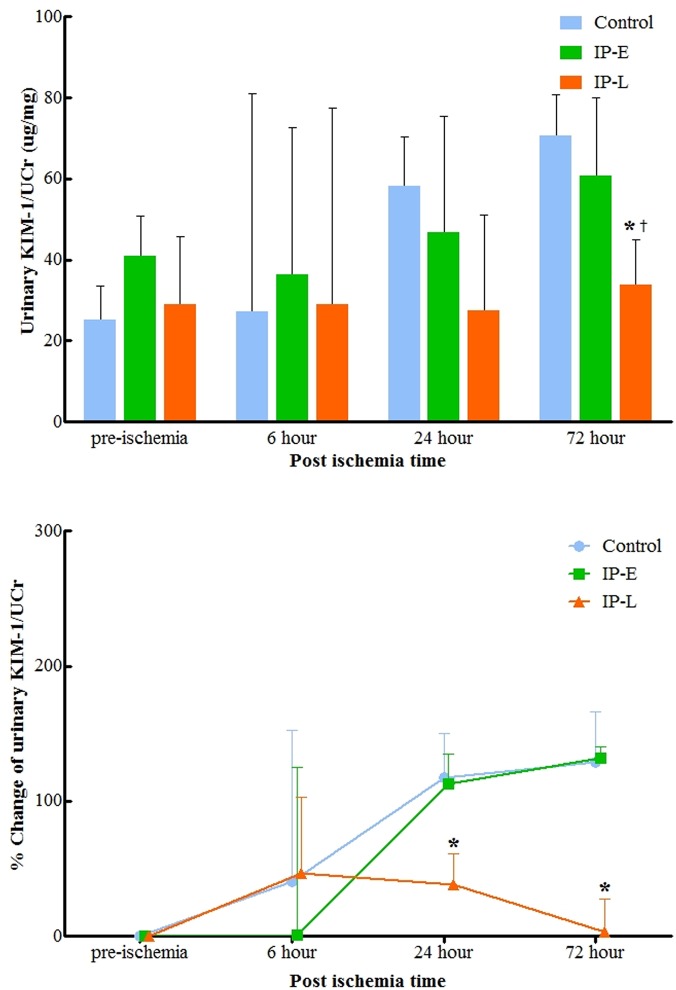
Changes in urinary KIM-1 (A) and urinary KIM-1 percent change from baseline (B) normalized to urinary creatinine. At 72 hours postoperatively, urinary KIM-1 was lower in the IP-L group than in the control and IP-E groups (p = 0.028 and 0.047, respectively). KIM-1, kidney injury molecule-1; Ucr, urine creatinine; IP-E, remote Ischemic preconditioning with early window; IP-L, remote ischemic preconditioning with late window. * p<0.05 vs. Control, ^†^ p<0.05 vs. IP-E.

Compared to NGAL, urinary KIM-1 levels exhibited a different pattern after IRI (Fig 4). The IP-L group exhibited very little change in KIM-1 levels throughout the experiment, whereas the control and IP-E groups showed continuous elevations of KIM-1 level. At 72 hours after ischemia, the urinary KIM-1 was lower in the IP-L group than in the control and IP-E groups (p = 0.028 and 0.047, respectively). At this time, KIM-1 in the IP-L group was almost at the pre-ischemic level, whereas the control and IP-E group KIM-1 levels were continuing to rise.

**Fig 5:**
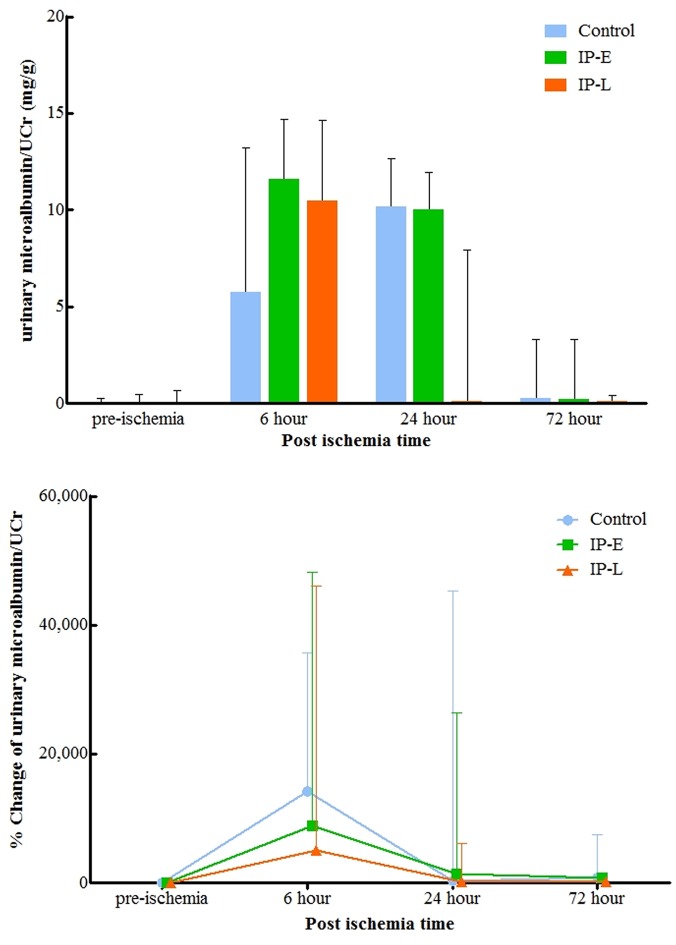
Changes in urinary microalbumin (A) and urinary microalbumin percent change from baseline (B) normalized to urinary creatinine. At 24 hours and 72 hours post-ischemia, urinary microalbumin tended to be lower in the IP-L group. However, no statistically significant differences were detected between the three groups until 72 hours after ischemia. UCr, urine creatinine; IP-E, remote ischemic preconditioning with early window; IP-L, remote ischemic preconditioning with late window.

Urinary microalbumin exhibited considerable changes throughout the observation period (Fig 5). All groups had substantially increased urinary microalbumin levels 6 hours after IRI, which did not differ between groups. At 24 hours and 72 hours post-ischemia, urinary microalbumin tended to be lower in the IP-L group. At 72 hours after IRI, the urinary microalbumin was lower in the IP-L group than in the IP-E group; however, the difference between groups was not statistically significant (0.21 [IQR 0.08–3.3] mg/g and 0.12 [IQR 0.07–0.44] mg/g, respectively; p = 0.079).

## Discussion

The use of IP has been demonstrated by numerous studies over the past 2 decades to have potent beneficial effects on preserving renal function after renal IRI [11]. However, despite these favorable results, IP has not yet been commonly used in humans, even for such procedures as renal transplantation or partial nephrectomy for renal tumor, which are representative clinical examples of renal IRI [24]. One of the reasons for the paucity of clinical use of IP is the lack of verification about the exact mechanisms of IP. Although previous studies have presented various potential mechanisms of IP [25–27], the mechanism of IP remains controversial and poorly understood. Another reason for the limited clinical use of IP for renal IRI in humans is the lack of standard protocols. We do not know which method is the most efficient to preserve renal function after IRI. There are a number of points to consider when establishing a protocol for IP, including the duration of clamping, the number of clamping cycles, whether to use an early window or a late window, whether to use local IP or remote IP, and which vessel to clamp if remote IP is chosen. The remaining uncertainly about these aspects of the process might lead clinicians to be hesitant about using IP for patients who will undergo partial nephrectomy or renal transplantation.

In their meta-analysis of 58 articles about IP in the animal kidney, Wever et al. concluded that SCr after IRI was lower in animals who had undergone renal IP than those without IP, as demonstrated in 62 experiments from 33 studies (standardized mean difference = 1.54, 95% confidence interval [CI] = 1.16, 1.93) [11]. However, 30 of these studies (91%) were performed in rats or mice. Rodents’ diets and renal physiology are very different from those of mammals, so it is extremely questionable to extrapolate these results to large animals or humans. The few studies examining the effects of IP in renal IRI in large animals have produced results that are quite different from those of the rodent experiments.

In a porcine experiment using local IP, Hernandez et al concluded that IP had no beneficial effect on either the SCr level or pathologic examination [13]. These authors measured serial SCr levels until 14 days after IP and renal IRI; they observed no differences in SCr between the IP and control groups throughout this period. This finding is comparable with our present results. In the current study, SCr also did not differ among the experimental groups. By contrast, levels of the renal injury markers, urinary KIM-1 and NGAL, were lower in the IP-L group than in the control and IP-E groups. KIM-1 and NGAL are two of the emerging biologic molecules that can indicate the exact degree of acute renal injury [28,29]. NGAL is a gene that is increasingly expressed during AKI [30]. KIM-1 is a protein that represents the degree of proximal tubular injury [31]. These markers can reflect kidney deterioration before the appearance of SCr changes [28]. Although not stated in the results, urinary NGAL at 24 hours was correlated with SCr at 72 hour in all experimental groups. Our findings thereby indicate that the IP-L group exhibited renal function preservation that was not reflected in the SCr. To our knowledge, this is the first study to measure renal injury markers in an IP experimental model.

The effects of IP-L on urinary NGAL and KIM-1 did not become apparent until 72 hours, postoperatively. It is not clear why these biomarkers did not show a difference earlier. In their study examining the mechanism of IP, Konstantinov et al. [32] verified that a remote IP stimulus modifies leukocyte inflammatory gene expression. The authors postulated that this effect might contribute to the protective effect of IP against IRI. When acute renal injury occurs, the initial inflammation occurs within the first three days, which consists of microvascular injury, obstruction, coagulation response, and tissue inflammation [33]. After that, renal tissue subsequently begins to de-differentiate, migrate, and proliferate. Taken together, remote IP might contribute to the initial inflammatory response of kidney, and thereafter, these effects appear after 72 hours, when the early phase of inflammation ends. Additional studies to further define the association between remote IP and renal inflammatory changes are necessary.

For cardiac IP, the concept of “second window of protection” has been previously described [34], which refers to the phenomenon in which protective effects reappeared at 24 to 72 hours after the acute effects had dissipated. The same phenomenon applies to renal IP. In their meta-analysis, Wever et al. [11] demonstrated that a late window (≥24 hours) of ischemia is more effective in reducing SCr after renal IRI than an early window (standardized mean difference = 2.43, 95% CI = 1.29, 3.57). However, few studies have directly compared the effects of an early versus late window of ischemia. In their experiments of local IP in dogs, Kosieradzki et al. [14] concluded that IP had no measurable effects on warm or hypothermic renal IRI in large animals, neither with early IP (10 minutes) nor late IP (24 hours) following 10-minute local preconditioning. These authors based their results on measurements of vascular resistance, glomerular filtration rate, urine production of the damaged kidney (compared with the contralateral normal kidney), and proximal tubular fluid reabsorption. However, concluding that IP with a late window of ischemia has no effect on renal IRI in large animals may not be valid because it has been demonstrated that local IP in large animals itself has no effect on renal IRI [13,16]. In another study, although using a mouse rather than a large animal model, Joo et al. [35] suggested different mechanisms for early window and late window of ischemia. The authors concluded that late window IP involves inducible nitric oxide synthesis, which is not involved in early window IP, and that genetic deletion or pharmacologic inhibition of inducible nitric oxide synthesis significantly reduces the protective effect of late window IP [35].

The present study has limitations, including its relatively small sample size, potential use of a less than the optimal IP time, and observations limited to only the early phase (first 72 hours) of renal IRI. For IP, the most effective duration of clamping for preconditioning has not been established. In the present study, the iliac artery clamping time was chosen as 10 minutes based on the existing literature [14,15]. Further study is necessary to definitively establish the optimal time period. It is regrettable that our observation period was limited to 72 hours, because the urinary renal injury biomarkers had begun to show differences at 72 hours postoperatively and SCr did not show any differences until 72 hours. However, Moon et al. [36] demonstrated that urinary NGAL levels decline earlier than SCr levels in recovery phase of AKI and that urinary NGAL can detect recovery from AKI. In addition, there was a limitation regarding the interpretation of the results, since the histologic analysis of renal tissues was not performed. Probably the most important limitation of this study was that it did not provide information about the mechanism of IP. More studies, such as those using microarrays of kidney tissue or reverse transcription polymerase chain reaction techniques, will be necessary to identify the mechanism of remote IP and to demonstrate why IP requires a time window of ischemia.

## Conclusion

While no protection was demonstrated, remote IP showed a significant reduction in biomarkers of renal injury in the porcine model when a time window of ischemia is used. Our study demonstrates that remote IP requires a considerable, rather than short, time window of ischemia before IRI to effectively provide organ protection. Before IP is used clinically in the field of human renal transplantation or partial nephrectomy, more studies about the optimal time window of ischemia and the exact mechanism of remote IP are necessary.
